# Close multilevel links between metabolic dysfunction-associated steatotic liver disease and type 2 diabetes mellitus

**DOI:** 10.1016/j.metop.2025.100424

**Published:** 2025-11-20

**Authors:** A.G. Holleboom, S.M. Francque, K. Cusi, C. Caussy

**Affiliations:** a Department of Vascular Medicine, Amsterdam UMC, the Netherlands; b Amsterdam Gastroenterology Endocrinology Metabolism Institute, Amsterdam UMC, the Netherlands; c Department of Gastroenterology and Hepatology, Antwerp University Hospital, Antwerp, Belgium; d InflaMed Centre of Excellence, Laboratory of Experimental Medicine and Paediatrics, University of Antwerp, Antwerp, Belgium; e Division of Endocrinology, Diabetes and Metabolism, University of Florida, Gainesville, Florida, USA; f Hospices Civils de Lyon, Département Endocrinologie, Diabète et Nutrition, Hôpital Lyon Sud, 69495, Pierre-Bénite, France; g Univ Lyon, CarMen Laboratory, INSERM, INRA, INSA Lyon, Université Claude Bernard Lyon 1, 69495, Pierre-Bénite, France

## Abstract

Since liver cirrhosis due to Metabolic Dysfunction Associated Steatotic Liver Disease (MASLD) was first described in patients with diabetes mellitus, the prevalence and severity of this liver disease have increased dramatically, driven by the increase in obesity and type 2 diabetes mellitus (T2DM). Strong epidemiological links between MASLD, insulin resistance and T2DM exist: T2DM is associated with higher prevalence and severity of MASLD, up to hepatic decompensation and hepatocellular carcinoma, and MASLD is in turn associated with incident T2DM Mechanistic studies support insulin resistance of metabolic tissues as the root cause of MASLD, whereas hepatic insulin resistance in turn contributes to hyperglycemia. Several pharmacological agents including incretin-based strategies, FGF21 analogues and the panPPAR agonist lanifibranor target the interface of MASLD and T2DM and have thereby shown promise to improve MASH and associated liver fibrosis. In light of the evident close multilevel links between MASLD and T2DM, care development efforts for MASLD in guidelines, local protocols and implementation strategies should aim to involve hepatologists, diabetologists, PCPs and their affiliated care teams in a joint effort to address the growing burden of fibrotic MASLD.

## Highlights

• Epidemiology links MASLD and T2DM bidirectionally: T2DM is associated with higher prevalence and severity of MASLD and MASLD is associated with incident T2DM.
• Mechanistic studies support insulin resistance of metabolic tissues as the root cause of MASLD, whereas hepatic insulin resistance in turn contributes to hyperglycemia.
• Pharmacological agents including incretin-based strategies, FGF21 analogues and the panPPAR agonist lanifibranor target the interface of MASLD and T2DM, thereby showing promise to improve fibrotic MASH.
• Care development for MASLD should involve hepatologists, diabetologists and PCPs to address the growing burden of fibrotic MASLD and T2DM.

## 1 Introduction

Liver cirrhosis from what we currently know as Metabolic Dysfunction Associated Steatotic Liver Disease (MASLD) was first described in 1938 in individuals with diabetes mellitus [1,2]. Since then, the prevalence of this disease, as well as the incidence of its severe cases have increased dramatically. This is driven by the global increase in obesity and type 2 diabetes mellitus (T2DM) in the obesogenic environment [[3], [4], [5], [6]]. Indeed, MASLD is currently the most prevalent chronic liver disease worldwide affecting approximately 30 % of the adult population [7]. In 2023, after a Delphi consensus process, the novel nomenclature metabolic dysfunction-associated steatotic liver disease (MASLD) was proposed to replace the term NAFLD. This multi-stakeholder initiative was motivated by the limitations encountered by the definition of NAFLD, based upon exclusionary criteria, and, most importantly, by the need to better reflect the underlying pathophysiology. The understanding of this pathophysiology has substantially improved over the last decade. An increasing amount of data also enabled a better apprehension of the broad disease spectrum of MASLD. This ranges from isolated hepatic steatosis with minimal risk of liver disease progression, to metabolic dysfunction-associated steatohepatitis (MASH) associated with an increased risk of liver fibrosis, cirrhosis and hepatocellular carcinoma (HCC) [8,9]. Based on all the progress made, improving clinical care is currently shaping up [[10], [11], [12]] to address the increasing number of patients with advanced stages of MASLD. These care implementation need to take into account the epidemiology of the MASLD spectrum, with a majority of patients with mild to moderate disease without significant liver fibrosis. In doing this, multidisciplinary approaches need to operate at the interface of hepatology and diabetology [11,13]. This is a crucial notion for both hepatologists, as MASLD is likely to dominate hepatology clinics for the foreseeable future, as well as for endocrinologists, as advanced fibrotic stages of MASLD represents a significant percentage of patients with T2DM. As the disease remains largely asymptomatic (or with unspecific manifestations such as fatigue that people do not link to liver disease) until the most severe stages such as cirrhosis decompensation or HCC, awareness and screening are necessary [11,12,14]. To this end, new guidelines and care initiatives aiming to guide implementation of this multidisciplinary approach are supporting healthcare givers and patients in addressing this major clinical need [[15], [16], [17], [18], [19], [20]].

In this review, we dissect the close interrelations of MASLD and T2DM at the interface of hepatology and diabetology into four levels: epidemiology, pathophysiological mechanisms, pharmacological development and development of multidisciplinary care.

## 2 Level 1. epidemiological links between MASLD, insulin resistance and T2DM

### 2.1 Prevalence of MASLD, T2DM and obesity

MASLD is an expression of insulin resistance which is associated with features of the metabolic syndrome [9,21,22]. Indeed, the first link between MASLD, insulin resistance and T2DM becomes clear from epidemiological studies. In the overall general population, the prevalence of MASLD has risen to a dramatic 25–33 %, making it currently the most prevalent chronic liver disease worldwide [7]. In the setting of obesity and/or T2DM, the prevalence of MASLD can rise to a staggering 65–80 % [7].

The increase in the prevalence of MASLD is closely linked to the global epidemic of obesity and metabolic diseases driven by sedentary lifestyle and excessive caloric intakes. Indeed, approximately 50 % of the patients with MASLD have obesity and up to 90 % of patients with severe obesity have MASLD [23]. Although obesity is also closely associated with T2DM, with an estimated prevalence reported up to 70–80 % in patients with T2DM in the United States [24], epidemiological studies highlight that MASLD, obesity and T2DM do not completely overlap [25]. For T2DM, in a meta-analysis comprising 2,241,753 individuals with T2DM, derived from 123 studies over 34 countries, the prevalence of MASLD was approximately 64.96 %, which hence was found to be twice as high as compared to the general population [7]. Furthermore, the prevalence of the active and potentially progressive stage of MASH has also increased over the past two decades and currently affects approximately 66 % of patients with T2D, whereas the prevalence of advanced MASLD-fibrosis is estimated to be approximately 15 % [7].

**Fig. 1:**
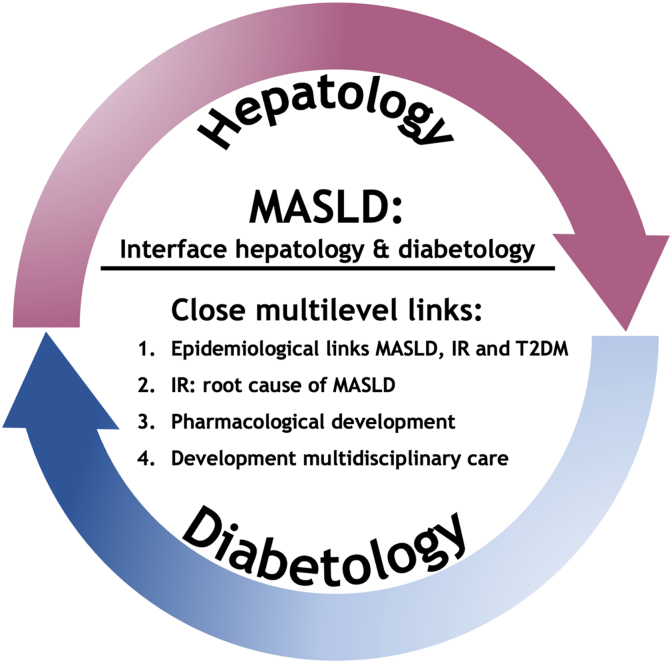
The close interaction between MASLD and T2DM at the interface between hepatology and diabetology dissected into four levels as detailed in this review. IR: insulin resistance.

Longitudinal studies and clinical outcomes related to MASLD and T2DM yield several important conclusions. First, they support the existence of a bidirectional (rather than diabetes being uniquely a causal factor for MASLD) relationship between T2DM and MASLD underlined by an increased incidence of T2DM in adult patients with MASLD [26] as well as in a paediatric population beyond the presence of obesity [27] (Fig. 1). And conversely, one study observed a decrease in incident T2DM when MASLD improves [28].

Secondly, an increased risk of macro- and micro-vascular complications in patients with both T2DM and MASLD compared to patient with T2DM without MASLD has been observed. The relative importance of both conditions has been well illustrated by the study from Ahlqvist et al. who performed hierarchical clustering in about 9000 newly diagnosed Scandinavian patients with diabetes classifying them into 5 different subtypes [29]. Interestingly, one subtype identified as the “severe insulin resistance diabetes (SIRD)” was significantly associated with the presence of steatotic liver as well as with an increased risk of coronary events and end-stage renal disease. These clusters have been further applied in the German Diabetes study and confirmed the association between the SIRD cluster and presence of hepatic steatosis [30].

Finally, an increased risk of progression towards advanced fibrosis as well as a higher risk of hepatic decompensation and HCC in patients with MASLD and T2DM compared to patients with MASLD without T2DM has been reported [31,32].

For both endocrinologists and hepatologists as well as other physicians seeing patients with MASLD such as general practitioners and preventive cardiologists, identifying patients with T2DM and MASLD enables to distinguish subpopulations with a higher risk of liver-related events including progression to cirrhosis, hepatic decompensation and HCC as well as patients with a higher risk of cardiovascular and renal events [14,33].

## 3 Level 2. insulin resistance as root cause of MASLD: mechanistic insights

**Fig. 2:**
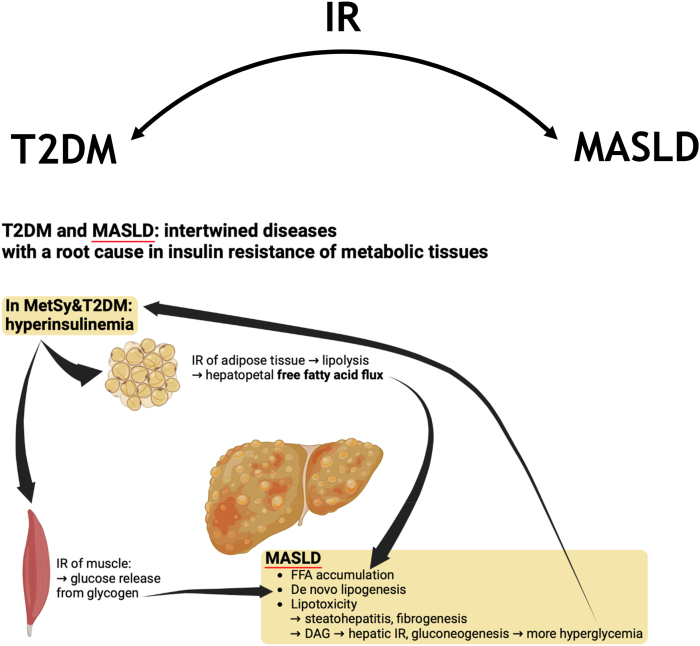
The reciprocal metabolic relation of T2DM and MASLD runs via mechanisms of insulin resistance.

The strong bidirectional epidemiological relations between MASLD and T2DM mentioned above are supported by reciprocal mechanistic links [21] (Fig. 2, Table 1). MASLD has its root cause in insulin resistance and the liver is a key organ involved in the regulation of glucose homeostasis [34]. Even lean patients with MASLD are invariably hyperinsulinaemic and insulin resistant, except for those rare cases with a genetic cause of steatotic liver disease such as a genetic VLDL secretion defect due to mutation in apolipoprotein B (familial hypobetalipoproteinemia) [35].

**Table 1:** Specific mechanisms of insulin resistance in adipose tissue, skeletal muscle and liver.

Metabolic tissue	Mechanism	Consequence	Effect on T2DM	Effect on MASLD	References
Adipose tissue	Lipolysis due to hypertrophy and oxidative stress	Hepatopetal free fatty acid flux	Hyperinsulinemia, beta cell stress	Major contribution accumulation fat in liver	[[39], [40], [41], [42], [43], [44]]
Skeletal muscle	Glucose release from excessive glycogen storage	Hyperglycemia and hyperinsulinemia	See consequence	De novo lipogenesis through ChREBP. LSEC dysfunction.	[[45], [46], [47], [48]]
Liver	Lipotoxicity, especially diacylglycerol	Translocation. protein kinase C ε from cytosol to the cell membrane, phosphorylation of hepatic insulin receptor, induction of hepatic insulin resistance	Increased hepatic gluconeogenesis	–	[[49], [50], [51], [52]]

Mechanistically, it has been hypothesized that in the setting of obesity, overeating and sedentary lifestyle, insulin resistance of muscle and peripheral white adipose tissue occurs as an initially appropriate cellular response mechanism against nutrient overload and mitochondrial oxidative stress when storage capacity for glycogen in the muscle and fatty acids in the adipose tissue is exceeded [[36], [37], [38]].

### 3.1 Insulin resistance of WAT

Yet when the insulin resistant state is present chronically and when adipose tissue hypertrophy causes physical stress and hypoxia [39,40], dysregulated lipolysis occurs in adipose tissue due to impaired phosphorylation of c-Jun N-terminal kinases (JNKs) and post-receptor insulin signaling [41]. Importantly, this brings about an increased free fatty acid (FFA) flux to the liver [42]. A study with metabolic fluxes and stable isotopes found that this FFA flux is the major contributor to intrahepatic lipid accumulation in MASLD: 59 %, versus 26 % from *de novo* lipogenesis (DNL) in the liver and 15 % directly from dietary sources [43]. In the insulin resistant state, this FFA flux is present continuously as a sign of metabolic inflexibility, driving MASLD [13,44].

### 3.2 Insulin resistance of skeletal muscle, hyperinsulinemia and *de novo* lipogenesis

In addition to this pathologic FFA flux derived from insulin resistant WAT, three complementary processes contribute to MASLD. Insulin resistance of skeletal muscle triggers glucose release from excessive intramuscular glycogen stores and this may contribute to DNL in liver, facilitated by the hyperinsulinaemia in patients with MASLD triggers hepatic signaling of sterol-responsive element binding protein 1c (SREBP1c) [45]. Of note, DNL rates are twice as high in patients with MASLD compared to the reference rates [46]. The hyperinsulinaemia may also be one of the triggers for dysfunction of the sinusoidal endothelium, inducing capillarization. In this process, liver sinusoidal endothelial cells (LSECs) lose their unique fenestrae and form a basement membrane, which may impact lipid trafficking thus promoting steatosis. LSEC dysfunction also occurs, characterized by reduced NO release which allows for activation of Kupffer cells and hepatic stellate cells, impacting on liver disease progression [47,48]. Thirdly, DNL especially increases with high fructose/sucrose consumption, which does not require insulin for hepatic uptake [53].

### 3.3 Lipotoxicity and hepatic insulin resistance

In conjunction with the occurrence of steatosis, hepatocytes themselves become resistant to insulin due to actions of lipotoxic subspecies, especially diacylglycerol (DAG) [49]. As elegantly demonstrated with two-step hyperinsulinaemic-euglycaemic clamps in combination with liver biopsies in patients with MASLD, DAG induces protein kinase C ε translocation from cytosol to the cell membrane, leading to phosphorylation of the hepatic insulin receptor and induction of hepatic insulin resistance, which in turn increases hepatic gluconeogenesis. Hereby, MASLD further increases hyperglycaemia in the state of IR/T2DM [50,51]. Recently Sabatini et al. demonstrated that glucose production is enhanced in individuals with MASH and is associated with hepatic fibrosis and inflammation. These findings reveal altered hepatic glucose metabolism, driven by hepatic and extrahepatic insulin resistance that contributes to the increased risk of hyperglycemia and T2D in individuals with MASH [52].

Taken together, evidence suggests a vicious reciprocal relation between MASLD and T2DM via mechanisms of insulin resistance of three main metabolic tissues.

## 4 Level 3. pharmacological treatment of MASH

Having synthetized the extensive evidence for the epidemiological and mechanistic links, a third clear link between MASLD and T2DM is observed in the development of pharmacological treatment for patients with MASH and significant fibrosis (stage 2 or higher).

### 4.1 Metabolic effects of investigational therapies for MASH

The development of effective treatments for fibrotic MASH is a highly active research field and multiple compounds in development for this indication target the metabolic interface between MASLD and T2DM [54,55]. Several of these molecules have demonstrated efficacy in resolving MASH and/or improving hepatic fibrosis in phase 2 randomized clinical trials (RCTs) and are currently being studied for their effectiveness and safety in phase 3 RCTs [56]. All of them are associated with favourable metabolic effects including improvement of at least one cardiometabolic factor, i.e. hyperglycemia, insulin resistance, obesity or dyslipidemia (Table 2).

**Table 2:** MASH therapies currently in development in phase 3 randomized controlled trials and their metabolic effects.

Class	Drug	Effect on HbA1c	Effect on insulin resistance (HOMA-IR)	Effect on body weight	Effect on lipid profile	RCT in MASH
THR-β agonist	resmetirom	neutral	neutral	neutral	↓ LDLc ↓TG, HDL-c: neutral ↓Apo-B	[57,138]
pan-PPAR agonist	lanifibranor	↓	↓	↑	LDLc: neutral ↓TG, ↑ HDL-C ↓Apo-B	[59,87,88]
GLP1-RAs	semaglutide	↓	↓	↓	LDLc: neutral or ↓ ↓TG ↑ HDL-c	[58]
Glucagon/GLP1 receptor dual agonist	survodutide	↓	↓	↓	↓ LDLc ↓TG ↑ HDL-C	[60]
FGF21 agonist	efruxifermin	↓	↓	neutral	↓LDLc ↓TG, ↑ HDL-C	[61]
	pegozafermin	↓	NA	neutral	↓ LDLc ↓TG ↑HDL-c	[62]

NA: data not available.

#### 4.1.1 Resmetirom

Resmetirom is the first FDA-approved drug for patients with MASH, as patients treated with resmetirom exhibited a significantly higher rate of both MASH resolution (up to 20.2 %, placebo-subtracted) and fibrosis improvement of at least one stage of fibrosis (up to 11.7 % placebo-subtracted) in the interim analysis of a large ongoing phase 3 RCT [57]. Resmetirom is a selective agonist of THR-β receptor in the liver, increasing beta-oxidation of fatty acids and lipid droplet turnover [57]. In line with its mechanism of action, resmetirom exerts no pronounced effects on glycaemic control, insulin resistance or body weight (Table 2). It is a well tolerated drug, with the main reported side effects being mild and gastrointestinal in nature. Its effects in compensated cirrhosis due to MASH are currently being investigated in an ongoing phase 3 trial (NCT05500222).

#### 4.1.2 Glucose-lowering effects of investigational therapies for MASH

In contrast to resmetirom, all other types of investigational therapies currently in phase 3 for fibrotic MASH affect glucose pathways (Table 2). These include the glucagon-like peptide-1 receptor agonist (GLP1RAs) semaglutide [58], the pan-PPAR agonist lanifibranor [59], the GLP1/glucagon co-agonist (survodutide) [60], and FGF21 analogues (efruxifermin and pegozafermin) [61,62].

Some therapies in development for MASH are currently approved for diabetes, GLP1RAs and glucose-dependent insulinotropic peptide (GIP)-GLP1 dual agonists and PPAR agonists. These drugs thus improve glycaemic control. Of note, they exhibited a higher efficacy for fibrotic MASH compared to resmetirom, with a placebo-subtracted effect ranging between 27 and 35 % for the proportion of patients with MASH resolution and from 14 to 20 % for the proportion of patients with liver fibrosis improvement of at least one stage of fibrosis (Table 2).

Due to the close relationship between insulin resistance, T2DM and MASLD, several anti-diabetic therapies, especially those known to improve insulin resistance, have been tested for MASLD improvement. Interestingly, some of these anti-diabetic treatments such as GLP1RAs mainly improve the extra-hepatic drivers of MASLD and MASH without a direct intrahepatic effect, underlining the importance of these extra-hepatic drivers in the pathophysiology of MASLD. However, targeting solely insulin resistance may not be sufficient to achieve significant improvement in MASH or liver fibrosis.

#### 4.1.3 GLP1RAs

GLP1 is an incretin hormone released by intestinal L-cells during the post-prandial phase. GLP1RAs act on GLP1 receptors expressed in several organs, such as the pancreas, intestine, adipose tissue, and brain. GLP1 helps regulate blood glucose levels by promoting insulin secretion in a glucose-dependent manner and suppressing glucagon release. Additionally, GLP1 contributes to weight loss via central effects on satiety and by delaying gastric emptying. The landmark phase 2 RCT with liraglutide reported a significant improvement in MASH compared to placebo [63]. Furthermore, in a substudy performed in 14 participants, the assessment of paired hyperinsulinaemic euglycaemic clamps, stable isotope tracers, adipose microdialysis and serum adipocytokine/metabolic profiling demonstrated a decrease in endogenous hepatic glucose production, an increase in insulin sensitivity and a decrease in hepatopetal FFA flux suggesting a decrease in *de novo* lipogenesis [64]. A larger phase 2 RCT with semaglutide using 0.1, 0.2 and 0.4 mg daily demonstrated a higher proportion of MASH resolution compared to placebo along with reduction of hepatic steatosis, but did not show significant improvement of liver fibrosis after 18 months of treatment [65].

Recently, the interim analysis of the ongoing phase 3 trial with semaglutide 2.4 mg once weekly showed significant improvement in both MASH and liver fibrosis, which may lead to conditional approval of semaglutide 2.4 mg weekly for the treatment of fibrotic MASH without cirrhosis [66]. Main side effect were nausea and diarrhea, a class effect for incretin-based therapies. While the safety profile of semaglutide 2.4 mg weekly was reported in a phase 2 RCT in patients with compensated MASH cirrhosis, no significant improvement of MASH or liver fibrosis was observed in this study population in a small RCT after 48 weeks [67].

The effect of GLP1RAs has been studied in large health insurance system databases. These retrospective studies underlined a significant reduction of risk of liver-related outcomes including incidence of HCC, liver transplantation and hepatic decompensation in patients with MASH-cirrhosis treated with GLP1RAs compared to non-GLP1RAs therapies [68]. A lower risk of progression to cirrhosis was also reported in patients with GLP1RAs therapy compared to DPP-4 [69] and no significant difference in incidence of hepatic decompensation was reported in patients treated with GLP1RAs compared to SGLT2i [70]. The second part of the ongoing phase 3 ESSENCE RCT with semaglutide 2.4 weekly in patients with MASH will provide additional information of the efficacy of semaglutide to reduce progression to cirrhosis and liver-related events [71].

#### 4.1.4 Dual glucagon/GLP1 receptor agonists

Survodutide, a dual glucagon/GLP1 agonist derived from glucagon is currently in phase 3 development for advanced fibrosis and compensated cirrhosis due to MASH. The dual agonism holds promise, because the extrahepatic benefits of GLP-1 receptor agonism (glucose control, reduced appetite, and weight loss) are combined with direct hepatic effects (increased energy expenditure, lipolysis, and mobilization of hepatic fat) associated with glucagon receptor agonism. The 48-week phase 2 trial with survodutide in 293 patients with F2-F3 MASH had 40 % T2DM. Average body weight reduction was 13 % and Hb1Ac reduction of 10 mmol/mol. The trial demonstrated a significant reduction of MASH and clear trends for fibrosis regression were observed. Main side effect were gastrointestinal [60]. Survodutide is currently in a phase 3 trial program for both fibrotic (NCT06309992) and cirrhotic MASH (NCT06632457). Within this subclass of dual glucagon/GLP1 receptor agonists, efinopegdutide [72] and pemvidutide [73] have also shown promise in Phase 2a trials for MASH using MR-based assessment of the liver, yet of note both compounds did not demonstrate reduction of HbA1C in these trials.

#### 4.1.5 Dual GIP/GLP1RA

Tirzepatide, a dual GIP and GLP1RA, which is expected to enter phase 3 development for fibrotic MASH, has demonstrated significant weight reduction, improvements in glycaemic control and insulin resistance, and reductions in hepatic, visceral and subcutaneous abdominal adipose tissue in patients with T2D and/or obesity in several clinical trials which has led to its approval for the treatment of T2DM and obesity [[74], [75], [76], [77], [78]]. In a phase 2 RCT for the treatment of MASH, tirzepatide demonstrated a higher proportion of resolution of MASH without worsening of fibrosis with a placebo-subtracted effect up to 52 % in patients with MASH and fibrosis stage F2 and F3. Additionally, a higher proportion of participant had an improvement of at least one stage of fibrosis without worsening of MASH with a placebo-subtracted effect up to 25 % [79]. There is no direct hepatic effect of tirzepatide and weight loss is considered as the main driver of liver improvement. Indeed, tirzepatide induces a greater magnitude of weight loss compared to semaglutide [80]. The precise role of GIP is debated, its activation in the adipose tissue enhances postprandial triglyceride uptake, increases free fatty acid oxidation and improves insulin sensitivity, which may contribute to the hepatoprotective effect [81,82]. The impact of tirzepatide on liver-related events is currently unknown, yet a large phase 3 trial which will evaluate this has been announced (NCT07165028).

#### 4.1.6 GLP1-GIP-glucagon triple agonist

Retatrutide, a GLP1-GIP-glucagon triple agonist, is in development for clinical obesity. Retatrutide is a single protein conjugated to a fatty diacid moiety. In a substudy of a 48-week RCT in obesity, steatosis regression in 98 participants with a liver fat content of ≥10 % at baseline was related to targeting metabolic dysfunction, evidenced by decreased body weight and abdominal fat, improved insulin sensitivity (as assessed with fasting insulin, fasting C-peptide and HOMA2-IR) and lipid metabolism and increased β-hydroxybutyrate as a sign of fatty acid oxidation (without inducing keto-acidosis). Of note, liver fat reduced rapidly, with a normalisation (<5 %) observed in 86 % of participants [83]. Retatrutide will be evaluated for outcomes in MASH in the same large phase 3 master protocol as tirzepatide (NCT07165028).

#### 4.1.7 FGF21 analogues

FGF21 analogues are associated with an improvement in glucose homeostasis and insulin resistance in pre-clinical mice models. However, these effects are not consistently observed in clinical studies, potentially explained by differences in the pharmacokinetics of the FGF21 analogues [84]. Both efruxifermin and pegozafermin are long acting FGF21 analogues currently in phase 3 development for fibrotic MASH and compensated MASH cirrhosis after having shown clear promise for fibrotic MASH [62,85] and in the case of efruxifermin also in compensated cirrhosis [86]. The trials with efruxifermin have consistently reported improvement in insulin resistance in patients with MASH fibrosis as well as cirrhosis [61,86]. However, the phase 1/2a and phase 2B RCTs with pegozafermin thus far showed more modest trends on glycemic parameters.

#### 4.1.8 Lanifibranor

A triple agonist from a different class is the PPARα, β/δ, and γ-agonist lanifibranor, which is in phase 3 development for fibrotic MASH after the phase 2 trial indicated regression of MASH and fibrosis [59] and improved cardiometabolic risk profile [87]. The phase 2 trial indicated an HbA1c reduction of 6 mmol/mol in highest dose group as the PPAR gamma effect reduced insulin resistance evidenced by a two-fold increase in adiponectin, on average inducing 3.1 percent body weight increase in the highest dose group as main side effect [59]. A recent study with hyperinsulinemic euglycemic clamps indicated lanifibranor indeed cuts the dysmetabolic engine of MASH, improving hepatic and peripheral (Rd) insulin sensitivity [88]. Its effect in cirrhosis are currently unknown.

### 4.2 Experimental combination therapy for fibrotic MASH including glucose-lowering compounds

As applied for other components of metabolic syndrome, *i.c.* dyslipidaemia, hypertension and T2DM, combination therapy for MASLD might be effective. Especially as some therapies mainly target extra-hepatic drivers of MASLD, enhanced therapeutic responses could be achieved in association with liver-targeted drugs. In this respect, semaglutide combined with the ACC inhibitor firsocostat and FXR agonist colifexor induced greater reductions in hepatic lipid content compared to semaglutide alone [89]. A post-hoc analysis of the phase 2 RCT of efruxifermin demonstrated a higher reduction in hepatic lipid content in patients treated with both GLP1RAs and efruxifermin compared to GLP1RAs alone [90]. Indeed, the combination of a GLP1RAs with a drug with a direct hepatic mechanism of action independent of weight loss, such as an FGF21 analogue, is appealing and is currently under investigation (NNC0194-0499) [91]. Furthermore, the small LEGEND trial in patients with MASLD and T2DM combined lanifibranor with the SGLT2i empagliflozin, showing clear reductions in HbA1c, hepatic steatosis and MRI-parameters of fibrotic MASH, in the absence of the weight gain observed in some patients treated with lanifibranor monotherapy (most likely attributable to the PPARу effect) [92]. Several other RCTs using a combination of drugs are currently conducted for the treatment of MASH (NCT04639414, NCT04321031, NCT04971785, NCT04065841, NCT05327127).

### 4.3 Lipid-lowering effects of investigational therapies for MASH

The drugs currently under investigation in phase 3 RCT for the treatment of MASH are often associated with improvement the profile of plasma lipids. The improvements in lipid profile may confer additional benefit, in particular reduction of cardiovascular risk associated with the decrease in LDL-c and non-HDL-c [93].

A significant decrease in LDL-c compared to placebo was observed with weight-neutral compounds in development for fibrotic MASH, i.c. resmetirom [57], survodutide [60] and efruxifermin [61] and pegozafermin [62], linked to their respective specific mechanisms of action. Resmetirom directly targets intrahepatic lipid metabolism. The activation of hepatic THR-β promotes the upregulation of genes involved in free fatty acid oxidation, lipoprotein catabolism, bile acids synthesis and increased expression of hepatic LDL receptors. Resmetirom reduces the production of very-low-density lipoprotein (VLDL), a precursor of LDL-c, which contributes to lowering LDL-c level [94].

The decrease in LDL-c observed with survodutide is linked to the hepatic effect of glucagon on lipid metabolism. Glucagon stimulates hepatic lipolysis and enhances cholesterol clearance from the circulation by stimulating the activity of hepatic LDL receptors [95].

FGF21 receptors are key regulators of lipid metabolism. FGF21 analogues stimulate fatty acid oxidation, inhibit *de novo* lipogenesis and decrease the VLDL export by downregulating the VLDL receptor expression in the liver. FGF21 analogues may also decrease LDL-c concentration by reducing cholesterol synthesis mediated by SREBP-2 inhibition, and stimulating cholesterol excretion mediated by enhanced bile acid synthesis in pre-clinical models [96].

### 4.4 Hepatic effects of standard-of-care glucose lowering agents

In addition to experimental therapies, many standard-of-care glucose lowering agents have been assessed for potential effects on MASLD often co-occurring with the T2DM. Most evidence here is retrospective or from smaller investigator-initiated trials (Table 3).

**Table 3:** Approved glucose-lowering therapies and their effects on MASLD.

Anti-diabetic therapy	Hepatic steatosis	MASH	Liver fibrosis		Liver-related outcomes Retrospective/observational data
			Fibrosis F1-F3	Cirrhosis (F4)	
**Metformin**	↔	↔	↔	?	*↓risk of HCC in cirrhosis*^a^ *↓risk of hepatic decompensation in cirrhosis*^a^
**DPP-4 inhibitors**	↔	?	?	?	↔
**Pioglitazone**	↓	↓	↔/↓	?	*↔* *↓risk of HCC*^a^*(?)* *↓risk of cirrhosis*^a^*(?)*
**SGLT2 inhibitors**	↓	↓?	?	?	*↓risk of HCC*^a^ *↓risk of cirrhosis*^a^ *↓risk of hepatic decompensation in cirrhosis*^a^
**GLP1RAs**	↓	↓	↓	↔	*↓risk of HCC in cirrhosis*^a^ *↓risk of hepatic decompensation in cirrhosis*^a^ *↓risk of cirrhosis*^a^
**GIP/GLP1 dual agonist**	↓	↓	↓	?	?

a Retrospective observational studies need validation in prospective trials.

### 4.5 Metformin

Metformin is the first line therapy for T2DM as it effectively improves hepatic and peripheral insulin resistance and inhibits hepatic gluconeogenesis [97]. Several RCT studied the hepatic effect of metformin and failed to report consistent significant changes in hepatic steatosis, MASH or liver fibrosis [98,99]. Metformin treatment in patients with compensated MASH-cirrhosis or biopsy-proven MASH with advanced fibrosis reported a lower cumulative incidence of need for liver transplantation or hepatic decompensation in retrospective observational studies [100,101]. In addition, metformin treatment in patients with T2DM was associated with a lower risk of HCC in a meta-analysis of 8 retrospective studies [102]. This suggests some benefit of metformin beyond short-term histological improvement, but these observations need validation in prospective trials.

#### 4.5.1 DPP-4 inhibitors

Dipeptidyl peptidase-4 (DPP-4) degrades endogenous GLP1 and GIP. DPP4 inhibitors indirectly promote GLP1 and GIP action through a longer half-life, thus leading to insulin secretion from beta-cells and decreased secretion of glucagon from alpha-cells. Few RCTs have investigated the effect of DPP-4 inhibitors mainly on hepatic steatosis with inconsistent effects and in small, usually open label or uncontrolled trials [103,104]. However, whether DPP-4 inhibitors improve MASH or liver fibrosis was not studied.

#### 4.5.2 SGLT2 inhibitors

SGLT2i decrease the glucose reabsorption by the kidney and are associated with a modest body weight reduction and decrease in blood pressure. SGLT2i′s mechanism of action is not mediated by an impact on insulin resistance and SGLT2i have no known direct effect in the liver. In an RCT including patients with well-controlled T2DM, empagliflozin treatment demonstrated a significantly higher decrease in liver lipid content compared to placebo but no change in insulin sensitivity assessed by euglycaemic-hyperinsulinaemic clamps was observed [105]. However, another RCT with patients with T2DM treated with dapagliflozin reported a significant decrease in hepatic lipid content associated with improvement of hepatic and adipose tissue insulin sensitivity assessed by euglycaemic-hyperinsulinaemic clamps [106] and this was also observed for canagliflozin [107]. Several other RCTs have consistently reported a significant decrease in liver lipid content in patients treated with SGLT2i compared to placebo or other anti-diabetic agents [105]. The mechanism behind the hepatic effect of SGLT2i is not fully elucidated. The improvement of hepatic steatosis is potentially at least partially mediated by the weight loss induced by calorie loss through increased glycosuria. Another mechanism may involve the increase in glucagon level and subsequent reduction of insulin/glucagon ratio, which could enhance fatty acid oxidation and inhibit *de novo* lipogenesis. Efficacy data of SGLT2i in improvement of histological lesion of MASH are scarce. Some evidence of improvement in some features of MASH, mainly hepatocellular ballooning, have been reported in a small open-label study with empagliflozin [108]. Three other open-label trials have reported a significant improvement of MASH and liver fibrosis with canagliflozin [109], ipragliflozin [110] and tofogliflozin [111].

Interestingly, recent retrospective case-control studies from large health system databases have consistently highlighted a lower risk of liver-related events including incidence of HCC and progression towards cirrhosis in patients with T2D treated with SGLT2i [112,113]. In patients with cirrhosis, the association of metformin and SGLT2i was also associated with a lower risk of hepatic decompensation and mortality [114].

#### 4.5.3 Thiazolidinediones

Thiazolidinediones are peroxisome proliferator-activated receptors (PPAR)-gamma agonists, which improve insulin resistance and lipotoxicity, especially in the liver, and increase lipid storage in subcutaneous adipose tissue. As a result of improved adipose tissue function, the secretion of adiponectin is increased while excess FFA release from insulin-resistant adipose tissue is decreased, which is thought to be the major driver of the hepatoprotective effect. A seminal RCT demonstrated a significant improvement in hepatic lipid content, hepatic insulin sensitivity and histological features of MASH in patients with impaired glucose tolerance or T2DM and biopsy-confirmed MASH treated with pioglitazone compare to placebo [115]. Additional RCTs have subsequently assessed the hepatic benefit of pioglitazone in patients with biopsy-proven MASH and consistently reported an improvement in both hepatic steatosis and MASH, also confirmed in a meta-analysis [116]. The effect of pioglitazone on liver fibrosis improvement is less clear with inconsistent results. Several meta-analyses have indicated a higher efficacy of pioglitazone compared to placebo in achieving improvement of at least one stage of liver fibrosis [117]. Another meta-analysis, including 8 RCTs, indicated that pioglitazone improved advanced liver fibrosis in patients with and without T2DM [118]. Combination therapy of pioglitazone and GLP1RA is associated with diabetes and overall metabolic improvement [119,120] and a reduction in hepatic steatosis and/or fibrosis [121,122]. The 2025 ADA has recommended consideration of the combination of pioglitazone with GLP1RA for the treatment of hyperglycaemia in adults with T2DM with MASH [123]. Saroglitazar is a double PPARα/y agonist approved for the treatment of T2DM and dyslipidaemia in India and has also recently been approved for treatment of MASH in this country [124].

The impact of long-term exposure of pioglitazone on liver-related events was not studied in prospective studies. Interestingly, observational data from population-based studies suggest that thiazolidinedione therapy in individuals with T2DM is associated with a reduced risk of developing cirrhosis [125,126].

## 5 Level 4. development of multidisciplinary care

### 5.1 Multidisciplinary approaches for management of MASLD and T2DM

As epidemiology, pathophysiology and investigational therapies indicate that MASLD and T2DM are twin diseases, adopting a multidisciplinary approach at the interface of hepatology and diabetology is an important task to reach an effective and scalable standard of care [13,127]. Indeed, this is clearly advocated by joint efforts of hepatology and diabetology societies [15,20,128,129] and facilitated by the development of clinical care pathways applying screening for MASLD in patients at cardiometabolic risk. Such pathways are based on algorithms of non-invasive test and proxies for advanced fibrotic stages of MASLD [12,130]. There is a striking agreement in these guidelines on the use of these non-invasive tests instead of liver biopsy to screen for advanced fibrotic MASLD. A FIB-4 of <1.3 can quite accurately rule out advanced fibrosis, and second-tier tests such as FibroScan® LSM >8.0 kPa or ELF™-score >9.8 can as well, in case of indeterminate FIB-4 [20,131]. In support, an elegant study demonstrated the effectiveness of such care pathway algorithms in improving identification of advanced fibrotic MASLD, whilst simultaneously reducing unnecessary referrals to hepatology clinics for early stages of MASLD [16]. Of note, this study was conducted in the setting of primary care providers (PCPs), not diabetologists. This puts forward the notion that PCPs should be involved in the interfacing care development for MASLD and T2DM, as they likely manage a large proportion of the patients with T2DM and metabolic syndrome who are developing advanced stages of MASLD. Another multi-centre study, performed in diabetology clinics, reported a good performance of the 2-step screening strategy for advanced fibrosis. This reinforces the applicability of such screening in individuals with T2DM [132]. Furthermore, a recent systematic review examining 9 care pathways studies for fibrotic MASLD in high-risk populations, comprising 11,566 patients, including 4 studies only in people with T2DM, concluded that the number needed to screen to detect advanced MASLD fibrosis was evidently lower in those with diabetes (n = 2–19) compared to screening the general population (n = 49) [133]. Screening for fibrotic MASLD may also be also warranted in preventive cardiology and nephrology, as cardiorenal risk is driven by identical cardiometabolic factors associated with an adverse disease course for MASLD. It becomes apparent that screening for fibrotic MASLD should be relevant to all health care professionals managing patients with obesity, T2DM and cardiometabolic risk factors. Accordingly, implementing such screening [134] requires flexibility regarding where screening tests are conducted. The setting may include diagnostic centres, GP practices by trained support personnel, diabetes nurses or nurse practitioners, or dedicated preventive hepatology clinics. The appropriate setting will depend on national or regional health care infrastructure specifics.

Important steps in reaching effective multidisciplinary approaches and care paths are establishment of national guidelines and adoption of these guidelines in local protocols at the level of hospitals and their adherence regions [12]. Together with these guidelines and protocols, this will take local championship from hepatologists and diabetologists in clinics with a focus on MASLD to raise awareness and knowledge in these clinics and their adherence regions [135], as well as support from scientific societies and governments. Major aiding factors in this process can come from the field itself. Better validation of the prospective value of NITs in large prospective cohorts will improve stratification of patients along the spectrum of MASLD and guide optimal follow-up. Increasing availability and reimbursement of NITs will also increase screening of patients at risk, with the example of access to transient elastography by the NICE in the UK [136,137]. The infrastructure of accessible diabetes care and preventive cardiology should be leveraged for implementation of preventive hepatology at scale, and effective co-management of T2DM and MASLD. This will allow nurses and nurse practitioners who treat non-communicable disease related to MASLD, with T2DM in particular to incorporate the central metabolic organ into their effective, guideline-based and scalable care in the next 10–15 years. Lastly, it is expected that approval of one or more pharmacological treatments for advanced fibrotic stages of MASLD will propel development of optimal care in the right direction.

### 5.2 Concluding remarks

Strong epidemiological links coexist between MASLD, insulin resistance and T2DM. Mechanistic studies support insulin resistance as the root cause of MASLD. Multiple pharmacological agents show promise to improve MASH and associated liver fibrosis by targeting the dysmetabolic interface of MASLD and T2DM. Future research can help define optimal combinations of these agents for synergistic anti-MASH effects.

Given the close multilevel links between MASLD and T2DM, developing clinical care pathways should involve hepatologists, diabetologists, PCPs and other related healthcare practitioners in a joint effort. Existing infrastructure of accessible diabetes care and preventive cardiology within different healthcare systems should be leveraged for implementation of preventive hepatology both at scale and for personalized approaches at the same time, enabling effective co-management of T2DM and MASLD.

Together, these developments have great potential to reduce disease progression of MASLD into advanced stages and prevent its dreaded complications.

## CRediT authorship contribution statement

**A.G. Holleboom:** Writing – review & editing, Writing – original draft, Investigation, Conceptualization. **S.M. Francque:** Writing – review & editing, Supervision. **K. Cusi:** Writing – review & editing. **C. Caussy:** Writing – review & editing, Writing – original draft, Conceptualization.

## Conflicts of interest

AGH received consultant fees from Echosens, Inventiva, Lilly, MSD and NovoNordisk.

CC received consultant fees from Gilead, NovoNordisk, AstraZeneca, Lilly, E-scopics, MSD, Bayer, Corcept and Echosens, grant support from Gilead.

SF's institution has received grants from Astellas, Falk Pharma, Genfit, Gilead Sciences, GlympsBio, Janssens Pharmaceutica, Inventiva, Merck Sharp & Dome, Pfizer, Roche. He has acted as consultant for Abbvie, Actelion, Aelin Therapeutics, AgomAb, Aligos Therapeutics, Allergan, Alnylam, Astellas, Astra Zeneca, Bayer, Boehringer Ingelheim, Bristoll-Meyers Squibb, CSL Behring, Coherus, Echosens, Dr. Falk Pharma, Eisai, Enyo, Galapagos, Galmed, Genetech, Genfit, Genflow Biosciences, Gilead Sciences, Intercept, Inventiva, Janssens Pharmaceutica, PRO.MED.

KC has received research support towards his university as principal investigator from Boehringer Ingelheim, Echosens, Inventiva, LabCorp and Perspectum; served as a consultant for Arrowhead, 89Bio, Boehringer Ingelheim, BMS, Dexcom, Echosens, Eli Lilly, GSK, MGGM, Novo Nordisk, Sagimet Biosciences, Terns Pharmaceuticals and Zealand Pharma.

CS Praha, Julius Clinical, Madrigal, Medimmune, Merck Sharp & Dome, Mursla Bio, NGM Bio, Novartis, Novo Nordisk, Promethera, Roche, Siemens Healthineers, Weatherden.
